# The Basolateral Nucleus of the Amygdala Executes the Parallel Processes of Avoidance and Palatability in the Retrieval of Conditioned Taste Aversion in Male Rats

**DOI:** 10.1523/ENEURO.0004-19.2019

**Published:** 2019-07-09

**Authors:** Tadashi Inui, Tomoaki Sugishita, Chizuko Inui-Yamamoto, Yasunobu Yasoshima, Tsuyoshi Shimura

**Affiliations:** 1 Division of Behavioral Physiology, Department of Behavioral Sciences, Graduate School of Human Sciences, Osaka University, Osaka 565-0871, Japan; 2 Department of Oral Anatomy and Developmental Biology, Osaka University Graduate School of Dentistry, Osaka 565-0871, Japan

**Keywords:** avoidance, conditioned taste aversion, new method of behavioral experiment, palatability, retrieval, the basolateral nucleus of the amygdala

## Abstract

Conditioned taste aversion (CTA) is an essential behavior for animal survival. Conditioned animals show avoidance and decreased palatability to a conditioned stimulus (CS) on CTA retrieval. In this study, we aimed to determine whether the basolateral nucleus of the amygdala (BLA) is involved in CTA retrieval and whether avoidance and palatability in CTA retrieval are processed in the BLA. We developed an experimental chamber for time-course analysis of the behavior to approach a spout and lick a CS. In this experimental chamber, we analyzed the behavior of male rats following microinjections of GABA_A_ receptor agonist muscimol or saline into the BLA. The rats showed two types of approach behavior: they either (1) approached and licked the spout or (2) approached but did not lick the spout. Muscimol injection into the BLA decreased the frequency of the latter type of approach behavior, indicating that BLA inactivation reduced avoidance to the CS. The muscimol injection into the BLA also significantly increased the consumption of the CS. Lick microstructure analysis demonstrated that intra-BLA muscimol significantly increased licking burst number and size, indicating that BLA inactivation attenuated aversion to the CS as large burst licking is an indicator of high palatability. These results suggest that the increase in CS consumption with intra-BLA muscimol injection was due to alterations in approach and aversive responses to the CS. Therefore, we conclude that the BLA plays an essential role in CTA retrieval by parallel processing of avoidance and palatability.

## Significance Statement

To understand the role of the basolateral nucleus of the amygdala (BLA) in the retrieval of conditioned taste aversion (CTA), we developed a new behavioral paradigm that overcomes the limitations of traditional behavioral procedures in CTA studies. In male rats, we measured time stamps of the approach behavior to a conditioned stimulus (CS) and licking of a spout. BLA inactivation not only increased CS consumption but also decreased avoidance and aversion to the CS. These results reveal that the BLA is essential for CTA retrieval by processing two distinct behavioral responses in parallel: avoidance and palatability. We further demonstrate that multi-dimensional analysis of animal behavior is an important factor for elucidating brain function.

## Introduction

Food can be contaminated with harmful substances. Malaise after eating contaminated food induces conditioned taste aversion (CTA) through the association between food and taste as a conditioned stimulus (CS) and malaise as an unconditioned stimulus (US). Conditioned animals show suppression of CS consumption, which is induced by avoidance (Parker, 2003), and decrease in palatability (Spector et al., 1988) to the CS on CTA retrieval.

The suppression of CS consumption on CTA retrieval is attenuated by microinjection of the benzodiazepine agonist midazolam or the AMPA receptor antagonist 2,3-dihydroxy-6-nitro-7-sulfamoyl-benzo[f]quinoxaline-2,3-dione (NBQX; Yasoshima and Yamamoto, 2005; Garcia-Delatorre et al., 2014). CS inhibits electrophysiological activities in 76% of recorded basolateral nucleus of the amygdala (BLA) units (Kim et al., 2010) and induces ERK activation in the BLA (Lin et al., 2010). However, there are controversial reports stating that pharmacological BLA inactivation may not impair CTA retrieval (Bahar et al., 2004; Garcia-Delatorre et al., 2010, 2014), and an intraoral infusion of an aversive CS does not alter Fos-like immunoreactivities in the BLA (Yamamoto et al., 1997). These contradictory findings suggest that more evidence is required to determine the role of the BLA in CTA retrieval.

We previously demonstrated that a CS activates the neuronal projections from the BLA to the nucleus accumbens core (NAcC), the anterior part of the bed nucleus of the stria terminalis (BNST), and the central nucleus of the amygdala (CeA; Inui et al., 2013). The projections from the NAcC to the ventral pallidum (VP) mediate decreased palatability of a conditioned aversion to saccharin (Inui et al., 2007, 2009, 2011; Inui and Shimura, 2014, 2017). The projections from the BLA to the BNST and the CeA exert an effect on fear and anxiety (Walker et al., 2009). Although the unique functions of these targets, NAcC, BNST, and CeA, suggest multiple roles for the BLA in behavioral expression, there is no evidence showing that the BLA is involved in either avoidance or palatability to a CS on CTA retrieval.

Previous studies on the BLA in CTA retrieval traditionally have employed either intake or taste reactivity tests. The intake test measuring consumed fluid volume provides direct evidence of voluntary fluid consumption and indirect evidence of avoidance and palatability because other factors, such as motivation and post-ingestive effects, can affect consumption. Although a taste reactivity test can evaluate palatability by analyzing animal oromotor responses to intraorally infused fluid (Grill and Norgren, 1978; Spector et al., 1988), investigating the avoidance response is unsuitable because the animals receive passive intra-oral fluid infusion. To overcome the limitations of these traditional behavioral procedures, we developed a new apparatus for multi-dimensional behavior analysis. This apparatus measures the pattern of licking displayed by animals during fluid consumption. The ingestive behavior for animals consuming fluids consists of sustained runs of rapidly occurring rhythmic licks, referred to as a burst, separated by pauses of varying lengths. Animals display different burst size (i.e., number of licks in a burst) between palatable and aversive taste solutions (Davis and Smith, 1992; Davis and Perez, 1993; Hsiao and Fan, 1993; Davis, 1996; Spector and St John, 1998; Monk et al., 2014). We applied these microstructural analyses of licking to evaluate palatability in animals drinking a CS voluntarily. The experimental chamber was also used to assess the avoidance produced by the establishment of CTA by measuring the approach behavior of how the animals attempted to come close to the CS by detecting the tip of the animal snout (Parker, 2003).

Using the experimental chamber and microinfusions of GABA_A_ receptor agonist muscimol into the BLA, we aimed to determine whether the BLA is involved in CTA retrieval and, if so, whether avoidance or palatability on CTA retrieval is processed by BLA neurons.

## Materials and Methods

### Subjects

Twenty-three male Wistar rats (CLEA Japan, Inc.) weighing 250–280 g on arrival were individually housed in a temperature- and humidity-controlled colony room with a 12/12 h light/dark cycle (7 A.M. lights on). Water and food (MF, Oriental Yeast Co., Ltd.) were available *ad libitum* in the home cage, unless otherwise specified. All animals were handled in accordance with the procedures outlined in the Guide for the Care and Use of Laboratory Animals (National Institutes of Health Guide), and all experiments were approved by the Institutional Committee on Animal Research of Graduate School of Human Sciences, Osaka University.

### Surgery

Following acclimation in the colony room for at least one week, guide cannulae were bilaterally implanted into the BLA of the rats. Under isoflurane anesthesia (induction 5%; maintenance 1.5–2%), their heads were fixed on a stereotaxic apparatus (SR-6, Narishige Scientific Instrument Laboratory). The scalp was incised, and the skull was exposed. Small holes were drilled above the targeted brain region. The coordinates were as follows: anteroposterior = –2.8 mm (posterior to bregma), mediolateral = ±5.0 (lateral to midline), dorsoventral = –7.5 mm (ventral to bregma). The guide cannulae (C315G, Plastics One Inc.) were lowered into the holes and secured into the skull using metal anchor screws and dental resin. After suturing of the wound, an analgesic (carprofen, 4.4 mg/kg, s.c.) and antibiotic (cefazolin, 0.5 mg/kg, i.m.) were administered. The rats recovered for at least one week with *ad libitum* water and food before the behavioral experiments.

### Apparatus

**Figure 1.**
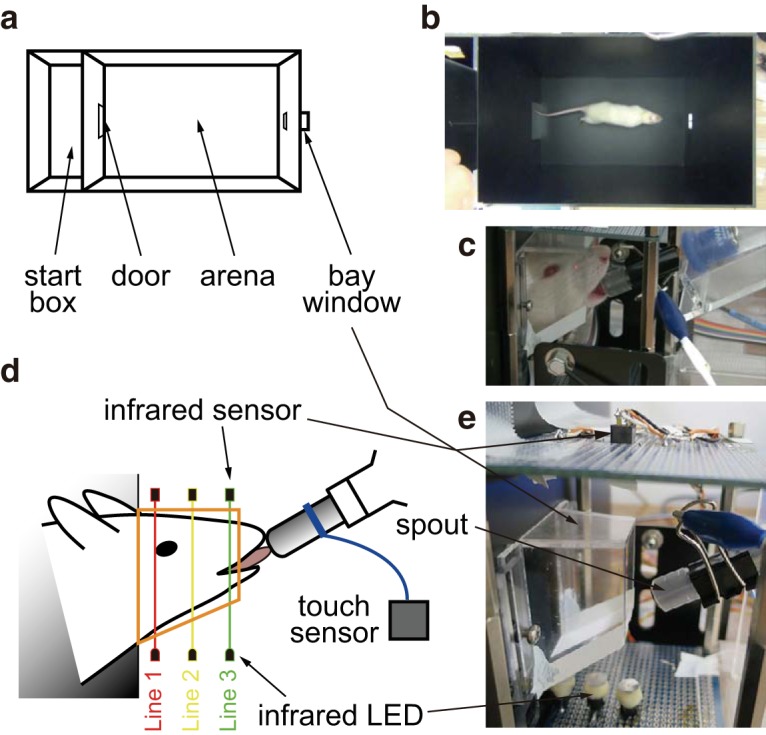
The experimental chamber. ***a***, The box was separated into a start box and an arena. Animals were put in the start box at the start of the trials. The animals were allowed to enter the arena through the door opened and closed by an experimenter. There was a bay window on the opposite side of the door in the arena. ***b***, The photograph shows a rat approach the bay window after the opening of the door at the start of a trial. ***c–e***, A diagram and photograph of the bay window. The rats put their heads into the bay window as shown in ***d*** to lick a spout in front of the bay window. Infrared LEDs below and sensors above the bay window detected the entry of the heads. The metal spout was connected with a touch sensor via a metal clip and wire.

All behavioral experiments were performed in a custom-made chamber (Fig. 1) that consisted of a start box, an arena, and a bay window (Fig. 1*a*). Animals were initially put in the start box (100 × 250 × 400 mm) and allowed to move to the arena (40 × 25 × 40 cm) through a guillotine door (100 × 100 mm) opened by an experimenter (Fig. 1*b*). The bay window was on the opposite side of the guillotine door. The size of the arena-side bay window was 50 mm high and 40 mm wide, while the outer-side of the box was 30 mm high and 40 mm wide, indicating that the bay window was tapered off and fitted to the shape of the rat’s head (Fig. 1*c*). There were three sets of infrared LEDs and infrared sensors that were parts of the passage detection sensor circuit (SY-852, Kyohritsu Electronic Industry Co., Ltd.) over and under the bay window (Fig. 1*d*, lines 1, 2, and 3). The transparent wall of the bay window allowed the infrared rays from the LEDs to reach the sensors (Fig. 1*e*). The three sets of LEDs and sensors were located at intervals of 2.5 cm. By analyzing which of the three sets of LED and sensors were shut off, we could determine the depth of entry of the rat’s head into the bay window. The shutting off of the infrared light between the LEDs and sensors triggered a square-wave pulse function generator (Akizuki Denshi Tsusho Co., Ltd.). A data logger (TUSB-S03CN3BZ, Turtle Industry Co., Ltd.) was used to collect the data of the square-wave pulses with a 10-Hz sampling rate that indicated the timing of entry of the rat’s head into the bay window.

In front of the bay window, a drinking tube was placed on a plastic holder. The drinking tube consisted of a metal spout and a conical tube (Fig. 1*d*,*e*). The metal spout was connected to a touch sensor circuit (SW-104, Kyohritsu Electronic Industry Co., Ltd.) via a metal clip and an electric wire. The touch sensor produced square pulse waves, but the current was too small to generate inputs to the data logger. Thus, the currents of the square pulse were amplified using an isolator (M2Y-6A-R2, M-System Co., Ltd.) and collected via the data logger for passage detection.

### Procedures of behavioral experiment

#### Habituation

The animals were acclimated to the chamber for 10 min on the first day in the start box. As the guillotine door was opened during the session, they could move in the chamber freely. The water deprivation schedule was started in the evening of the same day.

#### Training

Animals deprived of water for 20 h were initially put in the start box, and after 30 s an experimenter opened the guillotine door to allow the animals to move into the arena. After a rat completely moved to the arena, the guillotine door was closed. The session started at the opening of the guillotine door. The animals were allowed to drink water during a 30-min session. One hour after the end of the session, the rats were given food and water in their home cages until the next water deprivation period. This training was repeated for 4 d.

#### Conditioning

In this study, we expected the rats to retain aversion through the tests; we planned to examine the establishment of CTA on the first test and the effects of drug injections in second. We previously paired a CS with a US and found that some rats showed a rapid extinction of CTA after the first presentation of the CS (Inui et al., 2007). We needed rats to retain aversion through at least two test trials because we planned to validate the establishment of CTA on the first test and examine the drug effects on the second test. We also expected the conditioning procedure to not change the baseline of animal consumption behavior. In our preliminary study, we found that double conditioning after familiarizing with a CS is an ideal experimental procedure. Therefore, in this study, all the animals were pre-exposed to a 5 mM saccharin solution as a CS for three consecutive days (days 6–8), followed by two pairings of the CS with an intraperitoneal injection of 0.15 M lithium chloride (LiCl; 2% body weight) as a US on days 9 and 11. Five minutes after the end of the session, the US was administered in the home cage, and 2 h later, the animals were given ad lib food and water overnight for recovery. On days 10 and 12, water deprivation was re-started 20 h before the start of the next day sessions.

#### Test

The water sessions on days 13 and 14 assessed that the conditioning procedures did not cause changes in the baseline of animal consumption behavior. We confirmed the establishment of CTA on the first test (test 1, day 15). All the rats were microinjected with the vehicle (saline) into the bilateral BLA, 5 min before the start of the sessions. They were presented with the CS in the chamber. After test 1, the rats were divided into two groups that showed no significant differences in the CS intake (total number of licks) in the first conditioning (conditioning) test, test 1. In the second test (test 2, day 17), one of the groups received bilateral BLA microinjections of GABA_A_ receptor agonist muscimol, which inhibits neural activation. A control group was microinjected with saline. The experimental procedures of test 2 were identical to those used in test 1, except for the injected drugs.

### Microinjection of drug

Muscimol (M1523, Sigma-Aldrich Japan) was dissolved in sterilized saline (Otsuka normal saline, Otsuka Pharmaceutical Factory, Inc.) as a vehicle at a dose of 100 ng/0.5 μl. We used injector cannulae (C315I, Plastics One Inc.), which extended 1 mm from the tip of the implanted cannulae guide. The injector cannulae were connected to 10-μl of Hamilton gastight syringes (1701N; Hamilton Company) via polyethylene tubes (PE10; Becton, Dickinson and Company) and Viton tubes (JB_30_; Eicom Corporation) filled with Fluorinert (FC-40; Sumitomo 3 M). The syringes were mounted on a syringe pump (KDS210; KD Scientific). Muscimol or saline was injected at a rate of 0.25 μl/min for 2 min (total volume was 0.5 μl/side). The dose of muscimol was selected from previous studies, which showed inhibition of electrophysiological activity within a 1 mm radius of the injection area (Arikan et al., 2002) and impairment of behavioral performance (Wang et al., 2006; Piette et al., 2012).

### Behavioral analysis

**Figure 2.**
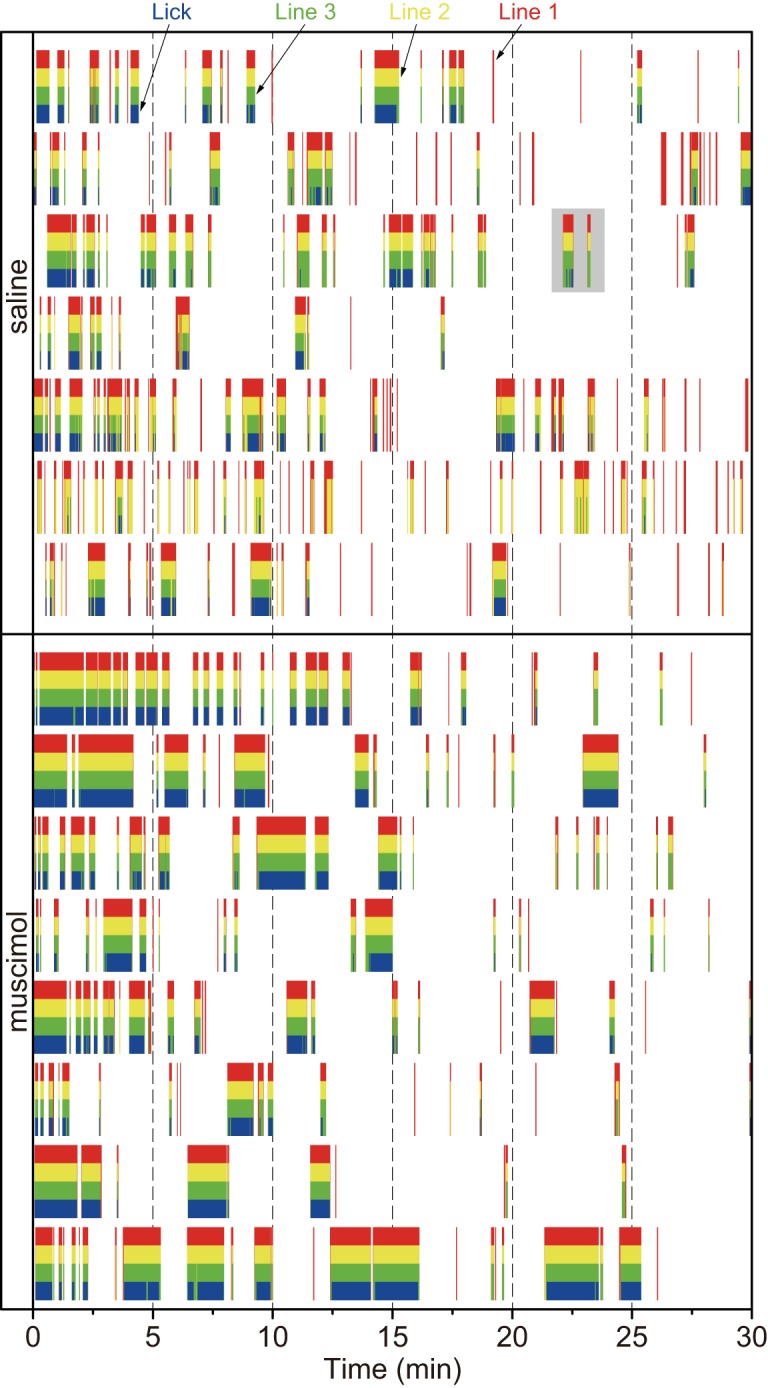
Raster plots of the responses of the passing and touch sensors in each animal on Test 2. The red, yellow, and green vertical lines indicate Line 1 (far from the spout), Line 2 (middle of the bay window), and Line 3 (close to the spout), respectively. The red color bars indicate that the rat’s head (tip of nose) stopped at the entrance of the bay window. Both the red and yellow color bars indicate the stop at the middle of the bay window, and the sets of the red, yellow, and green bars indicate the stop just before the spout. The plots with a gray background in the saline group are enlarged in Figure 3 for explaining behavioral analyses.

The outputs from the sensors were plotted in raster images (Fig. 2). These plots show both the entry of the rat’s head into the bay window and the licking pattern on an identical time scale. Based on these data, we categorized the approach and licking behaviors as follows.

### Entry

**Figure 3.**
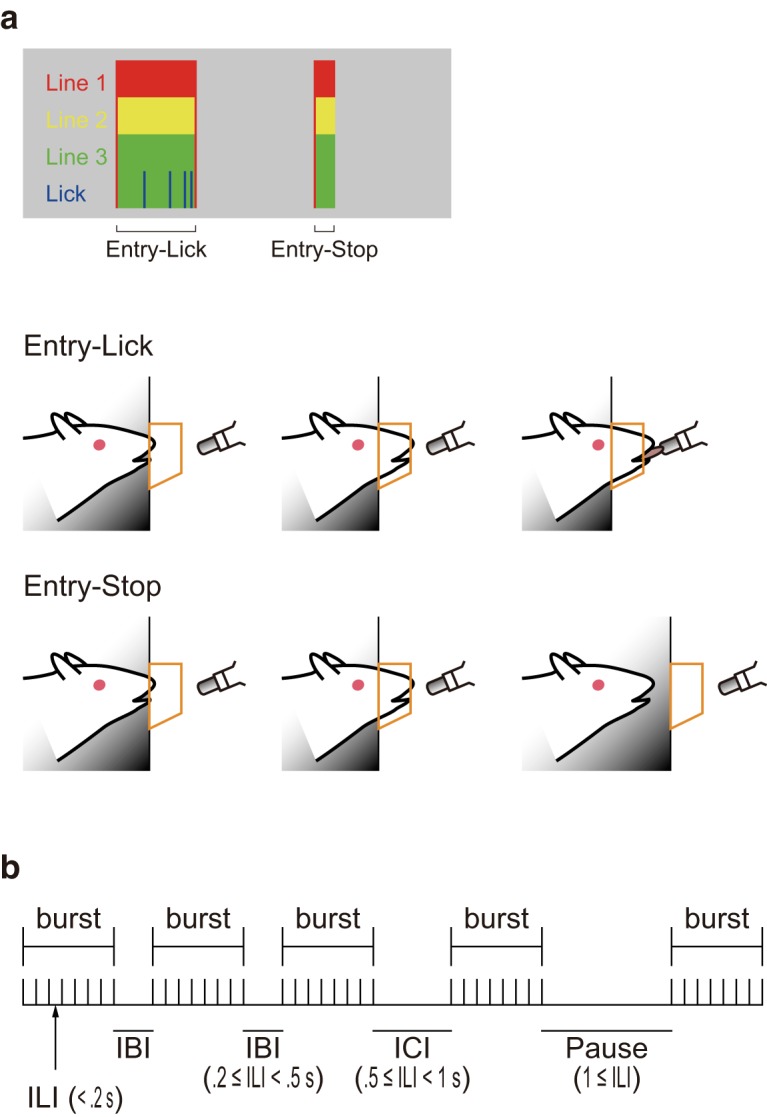
Scheme for behavioral analyses. ***a***, The analysis of the entrance of the rat’s heads into the bay window (Entry). Top, The blocks containing all the color bars indicate Entry-Lick, while the ones containing all the colors without blue indicate Entry-Stop. Bottom, Illustrations showing the animal’s behavior on Entry-Lick and Entry-Stop. ***b***, Schematic diagram of the licking pattern. The vertical lines indicate licks.

We defined lines 1, 2, and 3 as the combination of the infrared LEDs and sensors at the bay window (Fig. 1*d*), with Line 1 on the side of the arena. We also defined “entry” as the shutting-off, meaning that the tip of rat’s snout entered the bay window. The rats showed two types of entries (Fig. 3*a*). The entrance of their heads into the bay window with subsequent licking to the spout is referred to as the “entry-lick.” Alternatively, “entry-stop” was the event on which the rats entered their heads into the bay window but did not lick at the spout (their head stopped and stayed at the bay window).

### Licking

There are several lines of evidence, which suggest that microstructural analysis of animal’s licks to taste solutions reveal their palatability. Davis and Perez (1993) have defined “burst” as three or more licks with <250-ms interlick interval (ILI) between them. They showed that higher palatability of taste solutions produces increased number of bursts, burst duration (time from the first lick to last one in each burst), and burst size (number of licks in each burst). They have also defined interburst interval (IBI) as ≥250- and <500-ms ILI, and intercluster interval (ICI) as ≥500- or <1000-ms ILI, and pause as ≥1000-ms ILI. In this study, as the 10-Hz sampling rate produced a 100-ms time window, we have defined ILI as <200 ms, IBI as ≥200 and <500 ms, and other parameters were same as defined by Davis and Perez (1993; Fig. 3*b*).

### Data analysis

When the Shapiro–Wilk’s normality test revealed that the data of both groups (SAL and MUS) on each day (conditioning, test 1, and test 2) were normally distributed, the data were analyzed using a two-way repeated-measures ANOVA (group-day), with Tukey *post hoc* test where appropriate. When the Shapiro–Wilk test failed to show a normal distribution of the data, a nonparametric Mann–Whitney *U* test was used for the comparison between the groups. The within-subject comparisons were analyzed by a Friedman test, with a Wilcoxon signed-rank *post hoc* test. These statistical analyses were performed using the software OriginPro_2_016 (Origin Lab).

### Histologic analysis

We used fluorescent-conjugated muscimol (Muscimol, BODIPY TMR-X Conjugate, M23400, Thermo Fisher Scientific Inc.) to visualize the location of the tips of the injector cannulae. After completion of the behavioral experiment, the rats received an overdose of sodium pentobarbital (100 mg/kg). Thirty minutes before the overdosing, fluorescent-conjugated muscimol (250 ng/μl, 0.5 μl/side) was microinjected into the BLA. Transcardial perfusion of 0.02 M PBS was performed followed by fixation with 4% paraformaldehyde (PFA) in 0.1 M phosphate buffer. The brains of the rats were removed and post-fixed in 4% PFA overnight. The brains soaked in 30% sucrose in 0.1 M PB for cryoprotection were cut into 50-μm-thick slices and mounted on gelatin-coated glass slides covers slipped with Vectashield (Vector Laboratories). The locations of the injector tips were evaluated under a fluorescent microscope (AX70-80FLBD, Olympus Corporation) by an experimentally blind scorer.

## Results

### Location of cannulae tips

**Figure 4.**
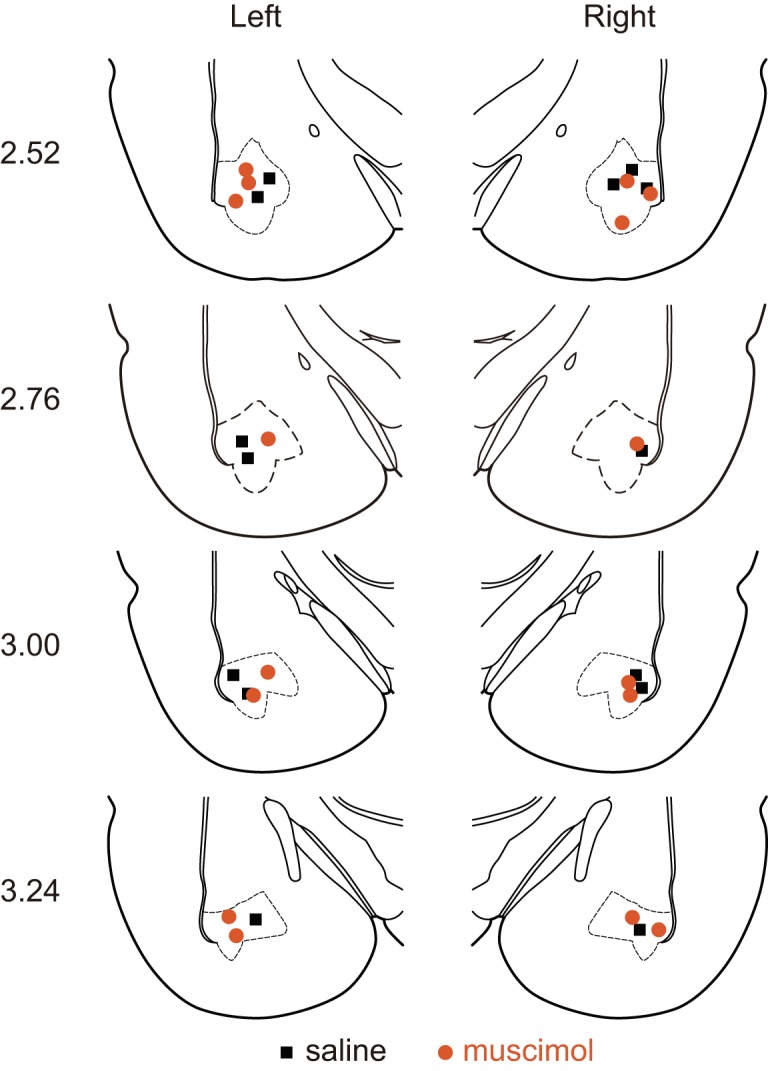
Histologic findings. The locations of the injection cannulae tips were reconstructed based on the Paxinos and Watson brain atlas (Paxinos and Watson, 2007). Orange circles, muscimol-injected rats; black squares, saline-injected rats.

Fluorescent images of the brain slices showed tissue labeled with red color around the track of the injector cannulae. We selected the slices having the brightest and largest labeled tissue as the center of the area for injections. Based on the selected slices, we constructed a stereotaxic mapping of the location (Fig. 4) modified from the brain atlas (Paxinos and Watson, 2007). They showed that eight MUS group rats and seven SAL group rats had the tips of the injector cannulae within the BLA. Eight rats (four muscimol-injected rats and four saline-injected rats) showed that either or both of the injector cannulae were out of the BLA. The behavioral experiment analyses excluded the data of these rats with the injector tips outside the BLA.

### Behavioral analysis

#### Entry

We analyzed the latency of the first entry and the frequency and duration of all entries irrespective of either entry-lick or entry-stop, to understand how the animals started to approach the bay window.

The latency of the first entry showed that the rats reached the bay window ∼5 s after the opening of the guillotine door on conditioning (Table 1). The pairings of the CS with the US did not change the beginning of the first approach on test 1. In test 2, while the SAL group showed about two times longer latency than test 1, the MUS group approached more quickly, though there was no significant group difference.

**Table 1.** Latency, frequency, and total duration of entry

	Conditioning		Test 1		Test 2	
	SAL	MUS	SAL	MUS	SAL	MUS
Latency of 1st entry	4.66 ± 0.84	5.91 ± 0.63	8.73 ± 0.93	4.18 ± 0.94	13.34 ± 4.27	4.46 ± 0.87^*^
Frequency of entry	57.43 ± 5.89	46.13 ± 4.86	41.43 ± 7.30	32.13 ± 4.02	67.86 ± 21.4	26.13 ± 2.91††**
Total duration of entry	534.6 ± 64.14	625.93 ± 35.99	243.19 ± 41.56	239.63 ± 41.57	255.67 ± 32.15	393.18 ± 62.49

** *p* < 0.01.

* *p* < 0.05 (vs SAL).

†† *p* < 0.01 (vs Conditioning).

**Figure 5.**
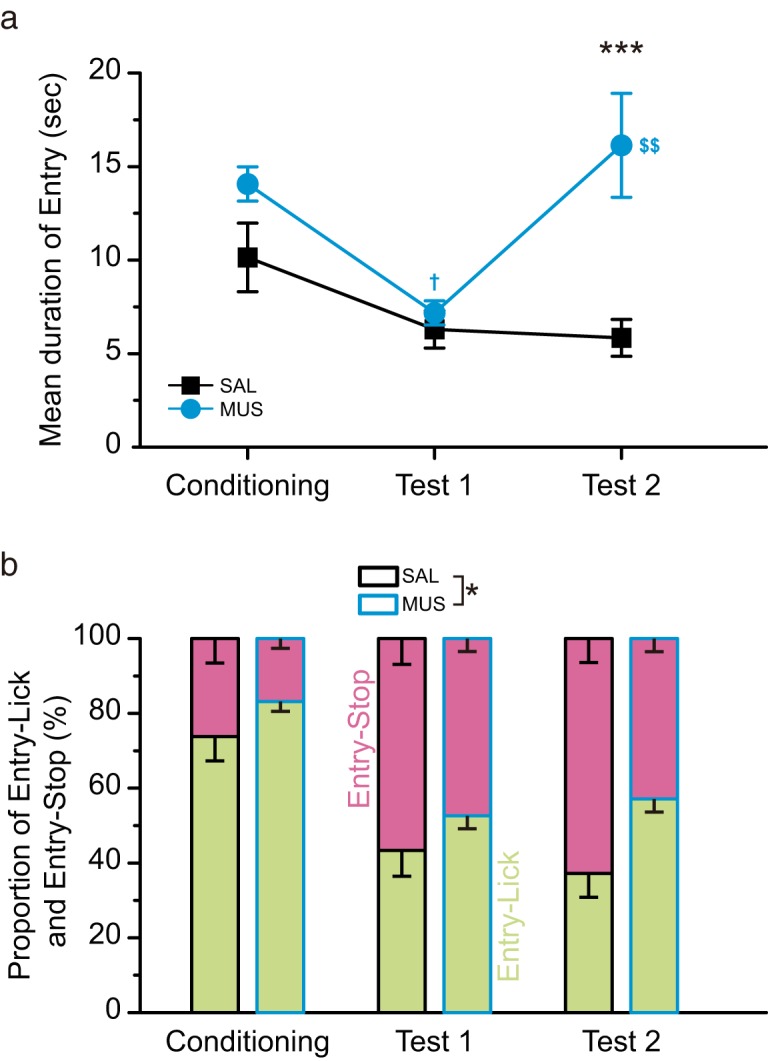
The intra-BLA muscimol altered Entry. ***a***, The mean duration of all Entry in the saline-injected (SAL) and muscimol-injected (MUS) groups on Conditioning, Test 1, and Test 2. After the CS-US pairings, the mean duration of Entry decreased in both groups. The microinjections of muscimol into the BLA on Test 2 impaired the decline in the mean duration of Entry. ***b***, The proportion of the frequency of Entry-Lick and Entry-Stop. The conditioning decreased Entry-Lick and increased Entry-Stop. The MUS group showed smaller proportion of Entry-Stop and larger Entry-Lick than the SAL group on Test 2; ***p < 0.001 (vs SAL), *p < 0.05, †*p* < 0.05 (vs Conditioning), $$*p* < 0.01 (vs Test 1).

The mean duration of entry, how long the rats stayed at the bay window through the sessions, significantly decreased after the CS-US pairings (Fig. 5*a*). The mean duration in the SAL group in test 2 was similar to test 1 and shorter than the mean duration in the Conditioning stage, while the mean duration in the MUS group for test 2 was longer than that for test 1 and was similar for the conditioning stage. A two-way repeated-measures ANOVA revealed significant main effects for group (*F*_(1,13)_ = 130.71, *p* < 0.001), day (*F*_(2,26)_ = 10.84, *p* < 0.001), and group-day interaction (*F*_(2,26)_ = 7.78, *p* < 0.01). A Tukey *post hoc* test showed a significant group difference in test 2 (*p* < 0.001). We found within-subject differences in the MUS group (conditioning vs test 1, *p* < 0.05; test 1 vs test 2, *p* < 0.01).

The frequency and total duration of entry decreased after the CS-US pairings in both groups (Table 1), which means the rats decreased approaches to the bay window. The analyses of the total duration of entry using a two-way repeated-measures ANOVA showed a significant main effect for day (*F*_(2,26)_ = 50.20, *p* < 0.001) but not a main effect of group (*F*_(1,13)_ = 1.89, *p* > 0.05) and group-day interaction (*F*_(2,26)_ = 2.08, *p* > 0.05). Conversely, a Mann–Whitney *U* test for the frequency of entry revealed a significant group difference for test 2 (Z = 2.49, *p* < 0.01). A Friedman test revealed that the differences in latency among trials were significant in the MUS group (χ^2^_(2)_ = 9.75, *p* < 0.01). A Wilcoxon signed-rank *post hoc* test indicated the frequency of entry for test 2 was significantly smaller than that for conditioning in the MUS group (*p* < 0.01). This suggests that the intra-BLA muscimol injections altered animal approach behavior.

We categorized entries into entry-lick and entry-stop and calculated the proportion of entry-lick and entry-stop frequencies (Fig. 5*b*). After the CS-US pairings, the proportion of entry-stop increased, while that of entry-lick significantly decreased. The MUS group showed a smaller proportion of entry-stop and a larger proportion of entry-lick than the SAL group for test 2. We analyzed the data of entry-lick and entry-stop using a two-way repeated-measures ANOVA, which revealed the main effects for group (*F*_(1,13)_ = 4.82, *p* < 0.05) and day (*F*_(2,26)_ = 53.87, *p* < 0.001) in both types of entry. The group-day interaction was not significant (*F*_(2,26)_ = 1.59, *p* > 0.05).

#### Entry-lick

**Figure 6.**
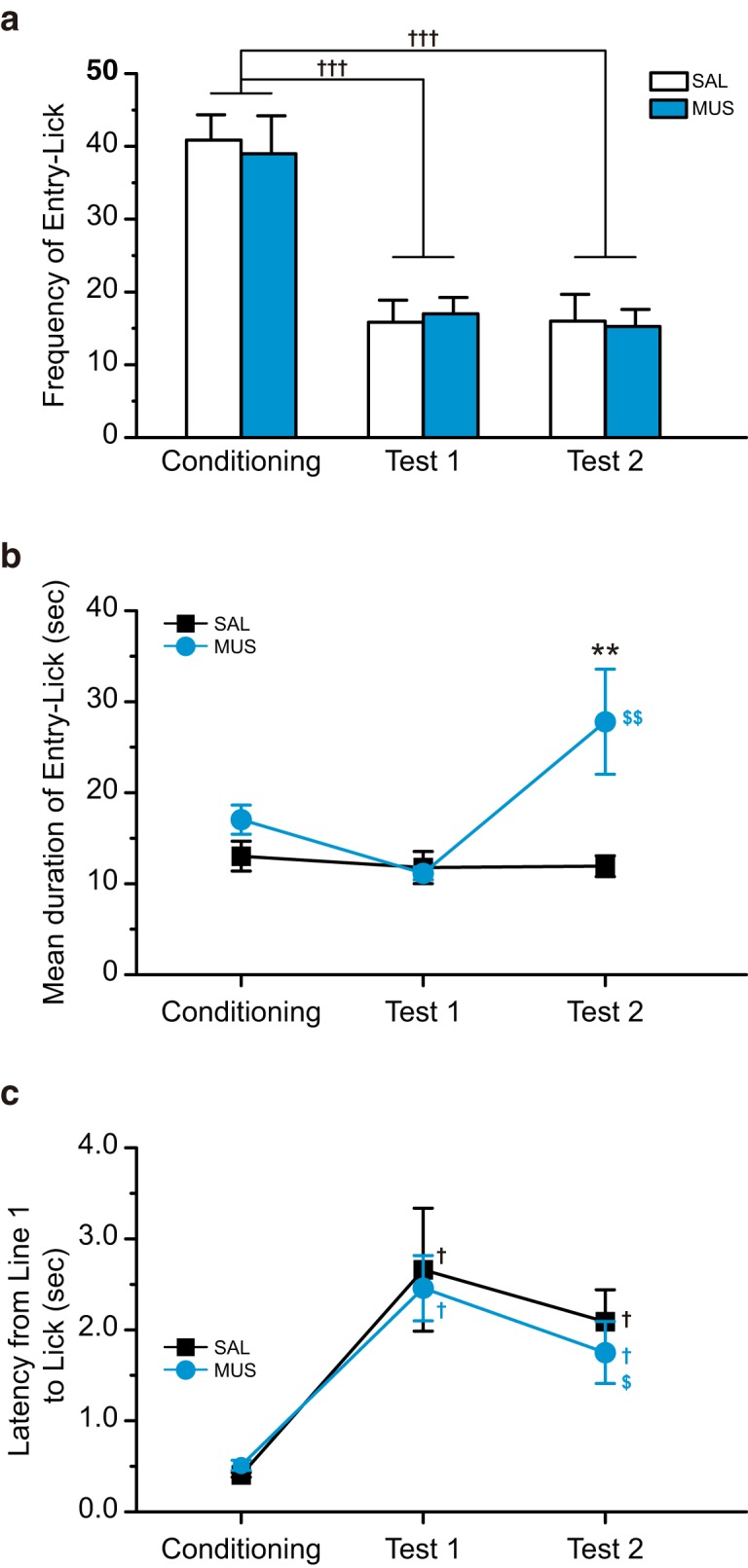
The intra-BLA muscimol decreased the mean duration of Entry-Lick. ***a***, The frequency of Entry-Lick in the saline-injected (SAL) and muscimol-injected (MUS) groups on Conditioning, Test 1, and Test 2. The CS-US pairings considerably decreased the frequency of Entry-Lick. Both the SAL and MUS groups showed suppressed frequency of Entry-Lick on Test 2. There was no group difference. ***b***, The mean duration of Entry-Lick. The rats showed a slightly shorter mean duration of Entry-Lick on Test 1 than Conditioning. On Test 2, the SAL group showed the same level of mean duration with Test 1, while the MUS group demonstrated significantly longer mean duration than Test 1 and the SAL group on Test 2. ***c***, The latency from the cut of Line 1 to Lick. The CS-US pairings significantly increased latency. The microinjections of muscimol into the BLA did not alter the latency in Test 2; **p < 0.01 (vs SAL), †††*p* < 0.001, †*p* < 0.05 (vs Conditioning), $$*p* < 0.01, $*p* < 0.05 (vs Test 1).

The CS-US pairings significantly decreased the frequency of entry-lick (Fig. 6*a*). The decreased frequency of entry-lick was also observed for test 2. A two-way repeated-measures ANOVA revealed a significant main effect for day (*F*_(2,26)_ = 50.75, *p* < 0.001). We found no significant main effect for group (*F*_(1,13)_ = 0.016, *p* > 0.05) and group-day interaction (*F*_(2,26)_ = 0.153, *p* > 0.05). A Tukey *post hoc* test showed that the frequency of entry-lick for test 1 and test 2 was significantly smaller than that for the conditioning stage in both groups (*p* < 0.001).

The mean duration of entry-lick was slightly decreased after the pairings of the CS-US (Fig. 6*b*). The MUS group showed an elevation of the mean duration on test 2. A two-way repeated-measures ANOVA revealed significant main effects for group (*F*_(1,13)_ = 4.78, *p* < 0.05), day (*F*_(2,26)_ = 6.42, *p* < 0.01), and group-day interaction (*F*_(2,26)_ = 6.52, *p* < 0.01). A Tukey *post hoc* test showed a significant group difference on test 2 (*p* < 0.01). The mean duration for test 2 was significantly longer than that for test 1 in the MUS group (*p* < 0.01).

We further analyzed the time needed to lick the spout after passing line 1 as the latency from Line 1 to lick (Fig. 6*c*), an index of the speed of head movements in the bay window. The latencies for test 1 and test 2 were larger than that for the conditioning stage. A Mann–Whitney *U* test showed no significant group difference in every trial (conditioning, test 1, and test 2). A Friedman test revealed that the differences in the latency among trials were significant in both groups (SAL, χ^2^_(2)_ = 10.57, *p* < 0.01; MUS, χ^2^_(2)_ = 14.25, *p* < 0.001). A Wilcoxon signed-rank *post hoc* test indicated significant differences between conditioning and test 1 (SAL, *p* < 0.05; MUS, *p* < 0.05) and conditioning and test 2 (SAL, *p* = 0.022; MUS, *p* < 0.05). We also found a significant difference between test 1 and test 2 in the MUS group (*p* < 0.001).

#### Entry-stop

**Figure 7.**
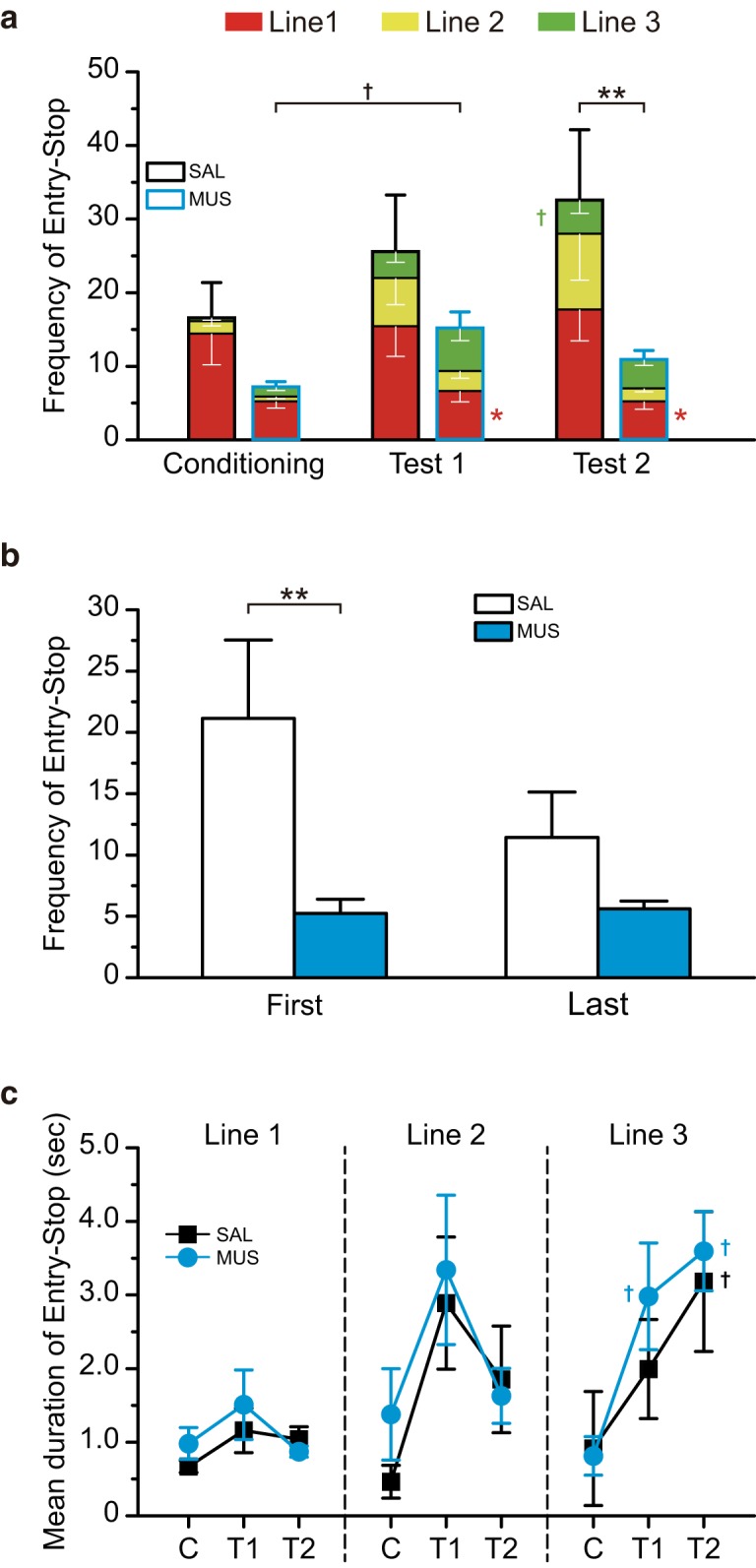
The intra-BLA muscimol decreased the frequency of Entry-Stop. ***a***, The frequency of Entry-Lick in the saline-injected (SAL) or muscimol-injected (MUS) group in Conditioning, Test 1, and Test 2. The CS-US pairings increased entry-stop. On test 2, the MUS group showed significantly smaller stops than the SAL group. ***b***, The frequency of Entry-Stop during the first and last half of Test 2. The muscimol-injected group showed smaller frequency of the stop on both the time windows. There was a significant group difference for the first half. ***c***, Mean duration of Entry-Stop at each line. The CS-US pairings significantly increased mean duration at lines 2 and 3. The mean duration at Line 2 decreased in both groups for Test 2 and increased at Line 3 for Test 2. However, there were no differences between the groups. C, Conditioning; T1, Test 1; T2, Test 2; **p < 0.01, *p < 0.05 (vs SAL), †*p* < 0.01 (vs Conditioning).

The CS-US pairings increased the frequency of entry-stop (Fig. 7*a*). For the SAL group, the frequency in test 2 was larger than in test 1, while the MUS group showed a decrease in the frequency. We analyzed data of the total frequency (sum of frequency at lines 1, 2, and 3) and the frequency at each line using a Mann–Whitney *U* test, which revealed significant group differences in total frequency for test 2 (Z = 3.01, *p* < 0.001). It also showed group differences in the frequency at line 1 for test 1 (Z = 2.04, *p* < 0.05) and test 2 (Z = 2.05, *p* < 0.05). The within-subject comparisons using a Friedman test showed significant differences among trials in the SAL group (line 3, χ^2^_(2)_ = 7.36, *p* < 0.05) and the MUS group (total, χ^2^_(2)_ = 6.94, *p* < 0.05). A Wilcoxon signed-rank *post hoc* test demonstrated significant differences in the frequency at line 3 between conditioning and test 2 in the SAL group (*p* < 0.05) and the total frequency between conditioning and test 1 in the MUS group (*p* < 0.05).

As the MUS group showed a lower frequency of entry-stop than the SAL group in test 2, we divided the session into a first and second half (Fig. 7*b*). MUS group demonstrated a significantly smaller frequency of entry-stop than SAL group in the first half (Z = 2.84, *p* < 0.01). This suggests that the intra-BLA muscimol injections significantly inhibited entry-stop on the expression of CTA.

The mean duration of entry-stop at lines 2 and 3 on test 1 increased after the CS-US pairings (Fig. 7*c*). Both groups showed a higher mean duration for entry-stop at line 3 than for conditioning. We analyzed the differences in mean duration at each line between the SAL and MUS groups using a Mann–Whitney *U* test. We found no significant group difference, indicating that the intra-BLA muscimol injections had no effect on staying time at the bay window. A Friedman test revealed significant differences in the mean duration among trials in both groups (SAL, χ^2^_(2)_ = 8.86, *p* < 0.05; MUS, χ^2^_(2)_ = 2.28, *p* < 0.05). A Wilcoxon signed-rank *post hoc* test showed a significant difference between conditioning and test 1 in the MUS group (*p* < 0.05) and between conditioning and test 2 in both groups (*p* < 0.05, respectively).

#### Licking behavior

**Figure 8.**
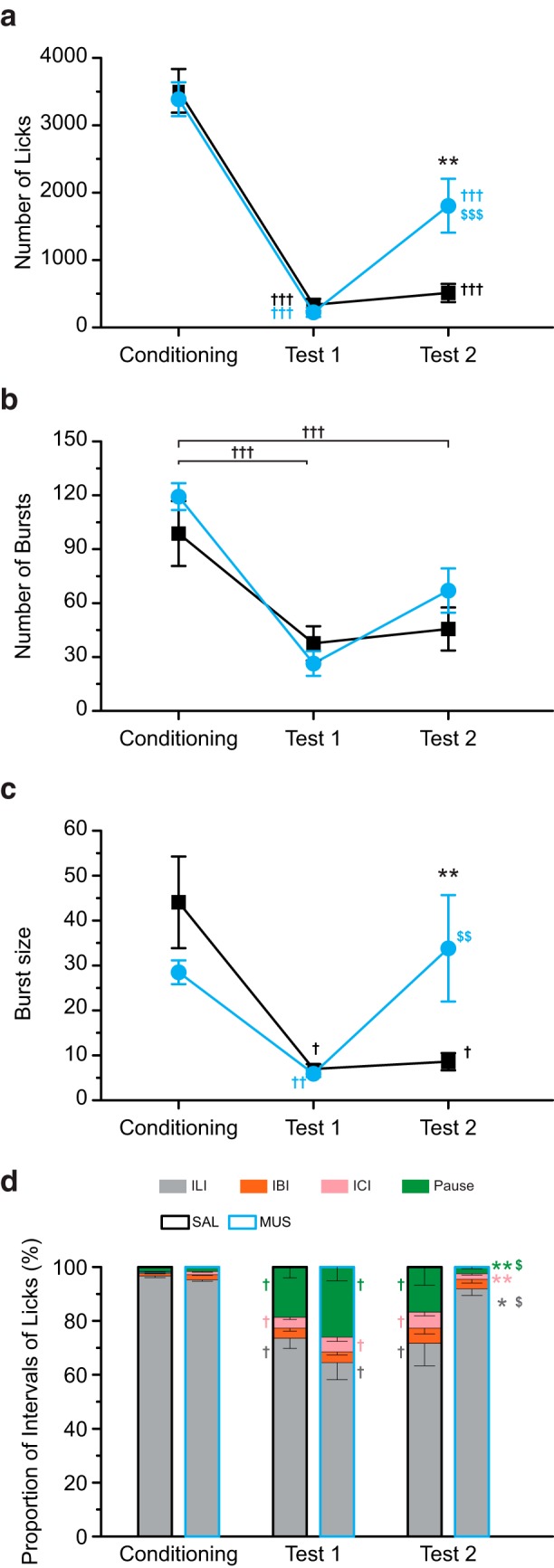
The intra-BLA muscimol attenuated aversion to the CS. ***a***, The total number of licks in the saline-injected (SAL) and muscimol-injected (MUS) groups on Conditioning, Test 1, and Test 2. The CS-US pairings significantly decreased the total number of licks. The MUS group showed larger CS licking than the SAL group on Test 2. ***b***, The number of bursts. The CS-US pairings decreased the burst number. There was no group difference for Test 2. ***c***, The size of the burst. The CS-US pairings decreased the burst size. The MUS group demonstrated a larger burst size than the SAL group for Test 2. ***d***, The proportion of ILI. The CS-US pairings increased the proportions of IBI, ICI, and pause, which were smaller in the MUS group than in the SAL group; **p < 0.01, *p < 0.05 (vs SAL), †††*p* < 0.001, ††*p* < 0.01, †*p* < 0.05 (vs Conditioning), $$$*p* < 0.001, $$*p* < 0.01, $*p* < 0.05 (vs Test 1).

The total number of licks during the session remarkably declined after the CS-US pairings (Fig. 8*a*). In test 2, the MUS group showed more licks than the SAL group. A two-way repeated-measures ANOVA revealed significant main effects for day (*F*_(2,26)_ = 149.43, *p* < 0.001) and group-day interaction (*F*_(2,26)_ = 9.28, *p* < 0.001). The main effect for group was not significant (*F*_(1,13)_ = 1.60, *p* > 0.05). A Tukey *post hoc* test showed a significant group difference for test 2 (*p* < 0.01). We also found the total number of licks in test 1 was significantly lower than in that in conditioning for both the groups (*p* < 0.001). In test 2, the SAL group showed a slight increase in the total number of licks (vs conditioning, *p* < 0.001; vs test 1, n.s.), while the MUS group had less lick number than in conditioning (*p* < 0.001) but larger than test 1 (*p* < 0.001). These results indicate that the intra-BLA muscimol injections increased CS licking but did not elevate it to the level before the establishment of CTA (conditioning).

After the CS-US pairings, the burst number largely decreased (Fig. 8*b*). There was no clear group difference in test 2. A two-way repeated-measures ANOVA revealed significant main effects for day (*F*_(2,26)_ = 35.71, *p* < 0.001). The main effect for group and group-day interaction were not significant (*F*_(1,13)_ = 0.73, *p* = 0.408; *F*_(2,26)_ = 1.99, *p* > 0.05). A within-subject comparison among the trials using a Tukey *post hoc* test showed significant differences in the number of bursts between conditioning and test 1 (*p* < 0.001) and conditioning and test 2 (*p* < 0.001).

The burst size also decreased after the CS-US pairings (Fig. 8*c*). In test 2, the MUS group showed a higher burst size. A Mann–Whitney *U* test revealed a significant group difference for test 2 (Z = –2.60, *p* < 0.01). A Friedman test showed significant differences among trials in the SAL group (χ^2^_(2)_ = 10.57, *p* < 0.01) and the MUS group (χ^2^_(2)_ = 13.00, *p* < 0.01). A Wilcoxon signed-rank *post hoc* test demonstrated a significantly small burst size for tests 1 and test 2 compared to conditioning in the SAL group (*p* < 0.05). On the other hand, the MUS group displayed a significant decrease in burst size for test 1 (*p* < 0.01) and increase for test 2 (*p* < 0.01).

**Figure 9.**
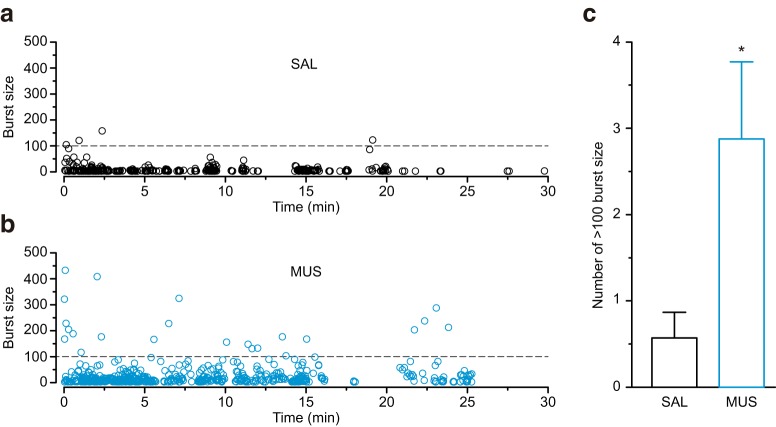
The intra-BLA muscimol increased larger burst size. Plots for all burst sizes in the saline-injected (SAL) and muscimol-injected (MUS) groups for Test 2. ***a***, The size of almost all the bursts was <100 licks in the SAL group. ***b***, The MUS group showed a larger number of lick bursts containing over 100 licks. ***c***, There was a significant group difference in the average number of >100 lick burst size; *p < 0.05 (vs SAL).

For a more detailed analysis of burst number and size, we plotted the size of all bursts in all the rats for test 2 (Fig. 9*a*,*b*). The MUS group displayed a significantly larger number of bursts with 100 licks more than the SAL group (*p* < 0.05; Fig. 9*c*). These results suggest that the intra-BLA muscimol injections increased burst licking to the CS.

The CS-US pairings altered the proportions of the ILI (Fig. 8*d*). The rats exclusively showed a <200-ms ILI in conditioning. In test 1, ILI decreased, whereas IBI (200 ≤ ILI < 500 ms), ICI (500 ≤ ILI < 1000 ms), and pause (1000 ms ≤ ILI) increased. The SAL group showed a similar proportion of intervals with test 1. Conversely, the MUS group had similar proportions with conditioning and demonstrated smaller proportions of ILI, IBI, and pause than in test 1. We analyzed group differences in the proportion of each type of interval using a Mann–Whitney *U* test. The MUS group showed significantly larger ILI (*p* < 0.05) and significantly smaller ICI and pause (*p* < 0.01, both) than the SAL group in test 2. A Friedman test showed significant trial differences in ILI (SAL, χ^2^_(2)_ = 10.57, *p* < 0.01; MUS, χ^2^_(2)_ = 13.00, *p* < 0.01), ICI (SAL, χ^2^_(2)_ = 11.14, *p* < 0.01; MUS, χ^2^_(2)_ = 7.75, *p* < 0.05), and pause (SAL, χ^2^_(2)_ = 10.57, *p* < 0.01; MUS, χ^2^_(2)_ = 12.00, *p* < 0.01). A Wilcoxon signed-rank *post hoc* test demonstrated significant differences in ILI, ICI, and pause between conditioning and test 1 (*p* < 0.05, all), conditioning and test 2 in the SAL group (*p* < 0.05, all). On the other hand, the MUS group showed no differences between conditioning and test 2, and significant differences in ILI and pause between test 1 and test 2 (*p* < 0.01, respectively). These results indicate that the establishment of CTA induced a longer ILI, and that intra-BLA muscimol injections made the ILI shorter.

## Discussion

We examined the effects of muscimol microinjections into the BLA on approach and licking behaviors in rats during CTA retrieval. We found that intra-BLA muscimol microinjection changes animal behavior, leading to an increase in the mean duration but not the frequency of entry-lick, a decrease in the frequency but not the mean duration of entry-stop, an augmentation of lick number and burst size, and a reduction in the ILI.

### Effects of muscimol injections on entry-lick

Intra-BLA muscimol increased the mean duration of entry-lick (Fig. 6*b*) but did not affect entry-lick frequency. The muscimol injections also induced a larger total lick number during the 30 min test session (Fig. 8*a*). As shown in Figure 2, the proportion of licking behavior for each entry (size of blue raster) was larger in the MUS group than in the SAL group. This indicates that muscimol injections induce relatively long-lasting licking behavior resulting in longer duration of entry-lick.

BLA inactivation reduces approach behavior to positive rewards, such as food and drugs (Fuchs and See, 2002; Kantak et al., 2002; McLaughlin and See, 2003; Fuchs et al., 2005; Rizos et al., 2005; McLaughlin and Floresco, 2007; Savage et al., 2007; Peters et al., 2008; Gabriele and See, 2010; Jones et al., 2010; Chaudhri et al., 2013; Parkes and Balleine, 2013; Parkes et al., 2016). Conversely, the entrance of the rat’s head into the bay window in this study was followed by licking to the aversive CS. Since the approach behavior was linked to a negative reward (e.g., CS), intra-BLA muscimol had no effect on the frequency of entry-lick. This also suggests that BLA inactivation does not cause changes in drinking motivation and locomotor activities.

### Effects of muscimol on entry-stop

Since intra-BLA muscimol decreased the frequency of entry-stop for test 2 (Fig. 7*a*,*b*), we examined why CTA establishment increases the mean duration of entry-stop, as shown in Figure 7*c*.

Several reports used a CTA paradigm as a model for approach-avoidance conflict (Weinberg et al., 1982; Brett et al., 1986; Ervin and Cooper, 1988). A conflict has been hypothesized to occur when two or more incompatible response tendencies compete (Miller, 1944). Simultaneous tendencies to approach and avoid the goal produce a conflict situation. In a single-bottle CTA study, when an animal is water-deprived and provided only with a conditioned aversive taste solution, the animal is in a presumed conflict situation between thirst (approach tendency, motivation to drink) and disgust (avoidance tendency, inhibition to drink; Ervin and Cooper, 1988). Since we put a spout in front of the bay window and the rats were water-deprived, the rats possibly were in an approach-avoidance conflict situation in test 1 and test 2.

Miller (1944) described that the degree of conflict depends on the relative strengths of the competing tendencies. Thus, maximum conflict arises when these strengths are equal. The animal’s proximity to the goal and drive state affects the strengths. The frequency of stops at line 3 (Fig. 7*a*) and mean duration of stops at lines 2 and 3 in test 1 (Fig. 7*c*) were larger than conditioning, indicating that the rats reached close to the spout (to line 3) but did not lick the spout even when they were motivated to drink fluid due to water deprivation after the CS-US pairings. Therefore, we assume that the entry-stop in test 1 and test 2 reflects a maximum approach-avoidance conflict situation.

The proportion of frequency for entry-stop in entire entry events for the MUS group was lower than that for the SAL group in test 2 (Fig. 5*b*). The SAL group tended to show a larger frequency for entry-stop in test 2 than in test 1, while the MUS group demonstrated no difference between test 1 and test 2. The frequency of entry-stop in the first half of the session in the MUS group was significantly smaller than the SAL group (Fig. 7*b*). These results suggest that intra-BLA muscimol reduces avoidance tendency. Since there were no changes in the frequency of entry-lick in the MUS group (Fig. 6*a*), the intra-BLA muscimol injections did not alter the approach tendency. Therefore, the reduction in avoidance tendency is considered to attenuate approach-avoidance conflict on CTA retrieval.

Intra-BLA muscimol had no effect on the mean duration of entry-stop (Fig. 7*c*) unlike on the frequency, indicating that the MUS group, similar to the SAL group, stopped and stayed in the bay window for a short duration. Since the lidocaine-reversible inactivation of the BLA was found not to affect spontaneous or basal locomotor activity (Woods and Ettenberg, 2004), the significant reduction in the frequency of entry-stop in the MUS group was unlikely to be associated with to the alteration in locomotor activities.

The administration of the anti-emetic ondansetron attenuates aversive taste reactions to the intra-orally infused CS, which is an index of decreased palatability, but it does not affect the consumption of the CS (Limebeer and Parker, 2000). They argue that animal behaviors on CTA retrieval comprise decreased palatability and avoidance. Therefore, the reduction in avoidance tendency in the MUS group in this study was possibly due to avoidance attenuation to the CS.

### Effects of muscimol on licking behavior

Intra-BLA muscimol increased the total number of licks (Fig. 8*a*), indicating increased CS consumption. Midazolam microinjections into the BLA increased the intake of the conditioned aversive sucrose (Yasoshima and Yamamoto, 2005). Midazolam does not directly activate GABA_A_ receptor but enhances the effect of GABA on GABA_A_ receptors, facilitating the opening of the Cl-channel. Thus, midazolam is considered to have a neural inhibitory effect. The administration of an AMPA receptor blocker NBQX into the BLA also increases the CS intake (Garcia-Delatorre et al., 2014). These findings indicate that the BLA inactivation might impair the suppression of the consumption of a CS on CTA retrieval.

The establishment of CTA decreases the burst number and size (Baird et al., 2005; Rebecca Glatt et al., 2016). The significantly reduced burst number (Fig. 8*b*) and burst size (Fig. 8*c*) in test 1 demonstrates that the rats in this study acquired a robust aversion. Although the group difference in the burst number in test 2 was not significant, the burst size in the MUS group was significantly larger than that in the SAL group. These results suggest that intra-BLA muscimol attenuated aversion to the CS.

The plots of burst size in test 2 (Fig. 9*a*,*b*) demonstrated a higher number of bursts with over 100 licks in the MUS group. These results suggest that intra-BLA muscimol tends to increase burst size. Higher concentrations of sucrose produce larger burst size (Davis and Smith, 1992; Davis and Perez, 1993; Spector et al., 1998), while conditioned aversive taste reduces burst size (Dwyer et al., 2008; Swick et al., 2015). Naturally, aversive quinine also produces smaller burst size than water (Spector and St John, 1998). Therefore, the burst size in the MUS group also suggests the attenuated aversion to CS by BLA inactivation.

The proportion of ILI (<200 ms) decreased, and that of IBI (200–500 ms), ICI (500–100 ms), and pause (>1000 ms) increased after the CS-US pairings (Fig. 8*d*). A previous study showed an increased proportion of longer ILI (Baird et al., 2005), supporting the argument that the rats in test 1 showed aversion to the CS. On test 2, the SAL group demonstrated proportions of IBI, ICI, and pause similar to those for test 1, whereas the proportions in the MUS group was similar to that in the conditioning stage. This indicates that attenuation of aversion to the CS by intra-BLA muscimol injections was also identified through the reduction in the ILI.

### The efferent projections from the BLA on CTA retrieval

CTA retrieval activates neuronal projections from the BLA to the NAcC (Inui et al., 2013) and NAcC neurons (Yasoshima et al., 2006) and changes the neurotransmission in the NAcC (Mark et al., 1991, 1995). CTA retrieval also activates GABAergic transmission from the NAcC to the VP (Inui et al., 2007, 2009). These findings suggest that the BLA-NAcC-VP neural circuits mediate the decreased palatability on CTA retrieval. Further, we have previously shown that CTA retrieval activates the neuronal projections from the BLA to BNST (Inui et al., 2013), but the role of the BNST in CTA retrieval is still unclear. Since the BNST is involved in fear responses (Davis, 1992; Duvarci et al., 2009; Walker et al., 2009), the BLA-BNST pathway may be involved in the avoidance tendency on CTA retrieval.

## Conclusion

In this study, we developed an experimental chamber for analyzing the temporal sequence of the behaviors of rat approaches to a spout and CS licking behaviors. The results showed that microinjections of muscimol into the BLA reduced avoidance to the CS. Muscimol injections also increased total CS licking, and lick microstructural analysis demonstrated that the attenuation of aversion increased CS licking. These results reveal that the BLA essentially contributes to CTA retrieval by simultaneous processing of avoidance and palatability to the CS.

## Synthesis

Reviewing Editor: Quentin Pittman, University of Calgary Decisions are customarily a result of the Reviewing Editor and the peer reviewers coming together and discussing their recommendations until a consensus is reached. When revisions are invited, a fact-based synthesis statement explaining their decision and outlining what is needed to prepare a revision will be listed below. The following reviewer(s) agreed to reveal their identity: Zoltán Karádi. Note: If this manuscript was transferred from JNeurosci and a decision was made to accept the manuscript without peer review, a brief statement to this effect will instead be what is listed below.

This manuscript deals with an important question regarding the role of the BLA in CTA retrieval. To that end the authors claim to use an original setup that allows them to measure both liquid consumption, lick bouts as well as approach behavior. They combined these measures to draw conclusions regarding the role of BLA in fear and palatability during CTA retrieval. From their results they conclude that the BLA contributes to CTA retrieval by processing both fear and palatability to the CS.

Despite the importance of the question studied, some aspects of the work weakens the interpretations stated by the authors. Concerns are listed in the points below.

Major:

1) Statistics: The authors have used two groups of rats, experiment and control, and recorded different behavior measures across 4 conditions. This design calls for statistical tests that will rely on the fact that the same rat was measured across conditions, such as repeated measure ANOVA, especially when the groups (SAL and MUS) were compared. However, the authors have used unpaired t-test multiple times to draw conclusions about the differences between the groups in Test2 (see for example page 21, lines 13-15). Repeated measure statistics is essential in the present experimental design because hypothetically there can be a bias in the groups that is hidden when they are combined, but is expressed when the two groups are separated. Additionally (but minor), there is a mixture of statistic presentations, where in some the p is equal to a value, and in others in is less or greater than a value. It is suggested to stick to one format.

2) The experimental design. The experiment described is far from regular, non-biased, CTA and thus drawing conclusions about the role of BLA in the specific time point is very problematic. For start, the rats went through familiarization to the saccharin for 3 days (days 6-8) prior to the conditioning protocol. This causes a latent inhibition effect (LI), which required 2 conditioning sessions instead of 1 which is done regularly. The authors briefly explained their incentive for performing LI (elimination of neophobia), but since what they study is retrieval, we do not understand their reasoning and why neophobia is a factor here. In the method section, page 11 line 11 they write: “It means novelty of taste can associate with visceral malaise”. But this is not the case. It is not the novelty of the taste that is being associated with the malaise, it is the perception of a novel taste, not novelty per-se. Moreover, even if neophobia is an important factor that should be eliminated, using sucrose (which does not elicit neophobic reaction) instead of saccharin would have worked. Second, Test1 was the first encounter with the taste after conditioning, and not surprisingly many measures reflected the aversion to the taste. Test2, however, has been done 2 days after the rats have experienced the taste without adverse effects, and thus extinction processes are known to kick in. That means that the results obtained in Test2 are a mixture of different experiences the rats have gone through (“saccharin is safe” > “saccharin is dangerous” > “maybe saccharin is safe?”). The interpretation of the BLA role in this scenario is difficult.

3) Interpreting Mus effect as the results of reduced fear. Although a valid interpretation of the results might be reduced fear directly, other interpretations exist that have not been ruled out. For instance, the reduced fear might be a consequence of attenuated memory of the association. If the BLA is part of the network that hold the association, inhibition of it might result an attenuated retrieval and wrongly interpreted as direct fear attenuation. (see for example Fortis-Santiago, Y., Rodwin, B. A., Neseliler, S., Piette, C. E., & Katz, D. B. (2010). State dependence of olfactory perception as a function of taste cortical inactivation. Nature neuroscience, 13(2), 158). Another option stems from the fact that your rats have initially learned that saccharin is safe (days 6-8). In this scenario BLA inhibition might promote extinction drive from other areas, such as from the mPFC. Moreover, fear is a feeling associated with danger or threat to the organism. We do not understand how refusal to approach the port might be regarded as fear from it. Fear is associated with several physiological changes such as fast heart rate etc. Perhaps when a rat understands that the spout contains the liquid it learned to hate, it does not show any clear fear responses. It not be appropriate to characterized avoidance behavior as fear. Last, even if the effect of the Mus is reduced fear, it might not necessarily be a reduced fear from the taste, but from the experimental chamber, as both conditioning and testing have been done in the same chamber. All in all , replacing ‘fear’ with “Avoidance” , which is what you measure would be a better alternative.

Minor:

1) The histological section in the methods section is not clear. It was not mentioned when the injection of the fluorescent-muscimol was injected. Was it the one in Test2 or later? Please clear this point. If the authors have used fluorescent-Mus it would be nice to know the spread of injection, since it might affect other areas. The figure currently show only tip location. And doesn't necessarily inactivate only the BLA.

2) In page 19 the first paragraph talks about “The duration of entry”, but in the second paragraph it said “The frequency and total duration of Entry significantly decreased ...”. What is the difference between “Duration” and “Total duration”?

3) We suggest using an English editing service. The English is not that bad, but can be improved.

4) The authors claim for a novel apparatus for better characterizing approach and avoidance behavior in CTA, however, the Davis rig has been used for many years in a similar manner. See for example “Monk, K. J., Rubin, B. D., Keene, J. C., & Katz, D. B. (2013). Licking microstructure reveals rapid attenuation of neophobia. Chemical senses, 39(3), 203-213” for a study of assessment of neophobia using licking structure.
